# Nonlinear Association of Estimated Pulse Wave Velocity With Bone Health Outcomes: Evidence From the NHANES Database

**DOI:** 10.1155/ije/4794146

**Published:** 2026-08-28

**Authors:** Xiaojin Zhu, Zhengjie Zhou

**Affiliations:** ^1^ Department of Orthopedics II, Pujiang County People’s Hospital, Jinhua 322200, China

**Keywords:** bone health, estimated pulse wave velocity, femoral neck bone mineral density, NHANES, nonlinear association, osteoporosis

## Abstract

**Background:**

The prevalence and incidence of impaired bone health have continued to rise globally, imposing a substantial public health burden. Cardiovascular‐related factors, including vascular aging, may be involved in the regulation of bone health. However, the association between the estimated pulse wave velocity (ePWV) and bone health outcomes remains unclear. This study aimed to investigate the relationship between ePWV and bone health using data from the National Health and Nutrition Examination Survey (NHANES).

**Methods:**

Data were derived from five NHANES survey cycles spanning 2005–2010, 2013–2014, and 2017–2018, comprising 13,068 eligible participants. Osteoporosis and femoral neck bone mineral density (BMD) were selected as bone health outcomes. ePWV was calculated using a validated formula based on age and mean arterial pressure. Descriptive statistics were used to summarize baseline characteristics. Group differences were assessed using nonparametric rank‐sum tests and Rao–Scott chi‐square tests. Multivariable weighted logistic regression models were applied to evaluate the association between ePWV and osteoporosis, while multivariable weighted linear regression models were used to examine the relationship between ePWV and femoral neck BMD. Restricted cubic spline (RCS) modeling was applied to assess potential nonlinear relationships, and subgroup analyses were conducted to assess effect modification across different population characteristics. Weighted receiver operating characteristic (ROC) analysis was performed to evaluate the discriminative ability of ePWV for osteoporosis, and percentile‐based trimming sensitivity analysis was conducted to assess the robustness of the findings.

**Results:**

Elevated ePWV levels were significantly linked to a higher likelihood of osteoporosis and reduced femoral neck BMD. In fully adjusted models (Model 3), each one‐unit increase in ePWV was associated with a 22% higher odds of prevalent osteoporosis (odds ratio (OR) = 1.22, 95% confidence interval CI: 1.14, 1.32, *p* < 0.001) and a decrease of 0.030 g/cm^2^ in femoral neck BMD (regression coefficients (*β*) = −0.030, 95% CI: −0.035, −0.024, *p* < 0.001). RCS and threshold effect analyses revealed significant nonlinear associations with identifiable inflection points between ePWV and both bone health outcomes. Subgroup analyses demonstrated generally consistent associations across most population subgroups, although effect heterogeneity was observed according to sex, age, body mass index (BMI), diabetes status, and alcohol consumption. Weighted ROC analysis showed moderate discriminatory performance of ePWV for osteoporosis, with an AUC of 0.774 and an optimal cutoff value of 8.865 m/s. Percentile‐based trimming sensitivity analysis yielded results consistent with the primary findings.

**Conclusions:**

ePWV is nonlinearly associated with osteoporosis and femoral neck BMD, and these associations appear to be robust across most population subgroups.

## 1. Introduction

Bone health refers to the ability of the skeleton to resist fractures and can be quantitatively assessed by measuring bone mineral reserves, including bone mineral content (BMC) and bone mineral density (BMD) [1]. Impaired bone health is commonly manifested as reduced bone mass, osteoporosis, and fragility fractures [2]. Among these indicators, BMD is the core quantitative measure of bone health, directly reflecting bone strength and load‐bearing capacity, and a decline in BMD represents a critical marker of bone loss [3]. As a systemic condition, osteoporosis involves the loss of bone substance and structural decay, affecting an increasing number of people on a global scale [4]. Epidemiological data indicate that osteoporosis affects nearly 200 million individuals worldwide, with postmenopausal women and older adults being at particularly high risk. These populations face a substantially increased lifetime risk of osteoporotic fractures [4, 5]. Beyond fracture susceptibility, osteoporosis is associated with chronic pain, functional impairment, reduced quality of life, and elevated risks of disability, prolonged immobilization, hospital readmission, and mortality, thereby imposing a significant burden on individuals, families, and healthcare systems [6, 7].

In recent years, growing evidence has suggested that cardiovascular‐related factors, particularly vascular aging, may be linked to bone health. For example, abdominal aortic calcification has been shown to correlate with decreased BMD and an increased risk of fractures at multiple skeletal sites [8]. Additionally, cardiovascular health indicators such as brachial pulse wave velocity have been reported to be inversely associated with BMD in women [9]. Several biological mechanisms may underlie the relationship between vascular aging and bone health. Increased arterial stiffness can elevate central pulse pressure and reduce microvascular perfusion within bone tissue, thereby disrupting nutrient delivery and bone metabolic homeostasis [10]. Moreover, vascular aging is often accompanied by chronic inflammation and oxidative stress, which may accelerate bone loss and compromise bone microstructure [11]. Collectively, these findings indicate that vascular aging–related markers may represent potential determinants of bone health beyond traditional risk factors.

Estimated pulse wave velocity (ePWV) is a surrogate index of arterial stiffness derived from age and mean arterial pressure, reflecting the propagation speed of the pulse wave through the arterial system [12]. Previous studies have demonstrated that pulse wave velocity increases with declining arterial elasticity and serves as a key indicator of vascular structural and functional aging [13]. Compared with carotid–femoral pulse wave velocity, which requires specialized equipment, ePWV offers the advantages of being noninvasive, easy to calculate, and highly reproducible, making it particularly suitable for large‐scale population‐based studies [14]. Nevertheless, evidence regarding the association between ePWV and bone health remains limited. Therefore, this study aimed to investigate the relationship between ePWV and bone health outcomes using data from the National Health and Nutrition Examination Survey (NHANES) database.

## 2. Materials and Methods

### 2.1. Study Population

This study utilized data from five cycles of NHANES: 2005‐2006, 2007‐2008, 2009‐2010, 2013‐2014, and 2017‐2018. The NHANES program operates as a continuous cross‐sectional study targeting the noninstitutionalized U.S. civilian sector to provide nationally representative data and is publicly available at https://www.cdc.gov/nchs/nhanes. The NHANES study protocols were approved by the Research Ethics Review Board of the National Center for Health Statistics (NCHS), and all participants provided written informed consent.

**FIGURE 1:**
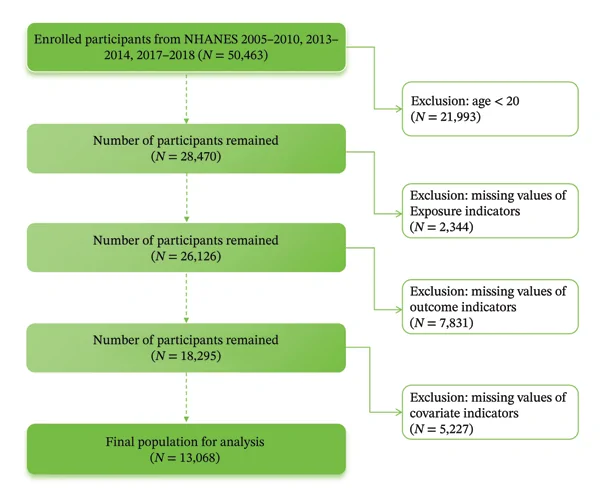
Flowchart of participant selection. Abbreviation: NHANES, the National Health and Nutrition Examination Survey.

A total of 50,463 participants were initially included. Individuals aged < 20 years and those with missing data on blood pressure, BMD, osteoporosis status, or relevant covariates were excluded. Ultimately, 13,068 participants were eligible for the final analysis. The detailed participant selection process is illustrated in Figure 1.

### 2.2. Calculation of ePWV

ePWV was calculated based on age and mean blood pressure (MBP). MBP was computed using the following formula [15, 16]:

(1) ePWV=9.5870.4024.5602.6213.1761.832−×age+×10−3×age2−×10−5×age2×MBP+×10−3×age×MBP−×10−2×MBP,

(2) MBP=diastolic  blood  pressure+0.4×systolic  blood  pressure−diastolic  blood  pressure.

### 2.3. Bone Health Outcomes

Two bone health–related outcomes were evaluated in this study: osteoporosis and BMD. BMD was measured using dual‐energy X‐ray absorptiometry (DXA) at the mobile examination centers with a Hologic QDR 4500A fan‐beam densitometer. All scans were performed by certified radiologic technologists following standardized protocols, and femoral neck BMD values were recorded.

Participants were excluded from DXA measurements if they had bilateral hip replacements, metal implants in both hips, a body weight exceeding 300 lbs (136 kg), pregnancy, or recent exposure to nuclear medicine examinations. Femoral neck T‐scores were calculated based on NHANES reference data using the following formula:

(3) T−score=participant’s BMD−mean BMD of the reference populationstandard deviation of the reference population.

Osteoporosis was defined using a composite criterion: (1) self‐reported physician diagnosis of osteoporosis and/or (2) a femoral neck T‐score ≤ −2.5 based on DXA measurements. This combined definition was applied to capture both self‐identified and clinically assessed cases of osteoporosis [17].

### 2.4. Covariates

A wide range of covariates were evaluated in this study, including age, sex, race/ethnicity, educational attainment, marital status, poverty–income ratio (PIR), body mass index (BMI), self‐reported history of diabetes, and cardiovascular disease (a composite variable including congestive heart failure, coronary heart disease, myocardial infarction, angina, and stroke). Lifestyle‐related covariates included smoking status (defined as having smoked at least 100 cigarettes during the lifetime), alcohol consumption (defined as consuming at least 12 alcoholic drinks in the past year), and moderate‐intensity physical activity (PA) (defined as engaging in moderate‐intensity exercise, fitness, or recreational activities for at least 10 min).

Laboratory variables included serum creatinine [18], gamma‐glutamyl transferase (GGT) [19], blood urea nitrogen, serum 25‐hydroxyvitamin D [20], and total serum calcium [21].

### 2.5. Statistical Analysis

All statistical analyses were conducted using R software (Version 4.4.2). A two‐sided *p* value < 0.05 was considered statistically significant. Descriptive statistics were used to summarize baseline characteristics of the study population. Continuous variables were expressed as medians with interquartile ranges (P25, P75), while categorical variables were presented as frequencies and percentages. To account for the complex, multistage sampling design of NHANES, nonparametric rank‐sum tests were used for continuous variables, and Rao–Scott–adjusted chi‐square tests were applied for categorical variables when comparing differences between participants with and without osteoporosis.

Multivariable regression models were constructed to evaluate the associations of ePWV with bone health outcomes. Model 1 was adjusted for age, sex, and race. Model 2 was further adjusted for PIR, marital status, education status, BMI, and PA. Model 3 included all covariates. Effect estimates were reported as odds ratios (ORs) or regression coefficients (βs) with corresponding 95% confidence intervals (CIs).

In the fully adjusted model, restricted cubic spline (RCS) functions were further applied to explore potential nonlinear associations between ePWV and bone health outcomes. Subgroup analyses and interaction tests were performed to assess whether the associations between ePWV and bone health outcomes differed across clinically relevant population strata. The subgroup variables examined in this study were selected based on biological plausibility and previous evidence linking demographic, metabolic, and lifestyle factors to arterial stiffness and skeletal health. Age, sex, BMI, alcohol consumption, diabetes status [22, 23], PA [24], and smoking status [25] were, therefore, included as clinically relevant stratification factors to explore potential heterogeneity in the associations between ePWV and bone health outcomes. Weighted ROC analysis was performed to evaluate the discriminative ability of ePWV for osteoporosis. The optimal cutoff value was determined by maximizing the Youden index, and the AUC, sensitivity, specificity, PPV, and NPV were reported.

To assess the robustness of the main findings, a percentile‐based trimming sensitivity analysis was performed to reduce the potential influence of extreme ePWV values. Specifically, participants with ePWV values below the 1st percentile or above the 99th percentile were excluded. The associations of ePWV with osteoporosis and femoral neck BMD were then reestimated using the same multivariable modeling strategy as the primary analyses.

## 3. Results

### 3.1. Baseline Characteristics of the Study Population

Baseline characteristics of participants stratified by osteoporosis status are presented in Table 1. Significant differences were observed between participants with and without osteoporosis across demographic characteristics, lifestyle factors, metabolic indicators, and bone health–related measures.

**TABLE 1:** Baseline characteristics of the study population stratified by osteoporosis status.

Characteristic	Overall, *N* = 13,068	Nonosteoporosis, *N* = 12,044	Osteoporosis, *N* = 1024	*p* value
Sex, *n* (%)				< 0.001
Female	6308 (49%)	5462 (47%)	846 (86%)	
Male	6760 (51%)	6582 (53%)	178 (14%)	
Race, *n* (%)				< 0.001
Mexican American	2220 (8%)	2101 (8%)	119 (4%)	
Non‐Hispanic Black	2367 (9%)	2267 (10%)	100 (5%)	
Non‐Hispanic White	6655 (73%)	5992 (72%)	663 (83%)	
Other race	1826 (10%)	1684 (10%)	142 (8%)	
Age, years	48.00 (37.00, 60.00)	47.00 (36.00, 59.00)	67.00 (57.00, 76.00)	< 0.001
Marital status, *n* (%)				< 0.001
Married/living with partner	8183 (67%)	7655 (67%)	528 (57%)	
Never married	1887 (14%)	1838 (15%)	49 (4.1%)	
Widowed/divorced/separated	2998 (19%)	2551 (18%)	447 (39%)	
BMI (kg/m^2^)	27.40 (24.00, 31.40)	27.50 (24.10, 31.50)	25.60 (22.70, 29.70)	< 0.001
Education status, *n* (%)				< 0.001
Above high school	3351 (17%)	3062 (16%)	289 (20%)	
High school	3069 (23%)	2793 (23%)	276 (27%)	
Under high school	6648 (60%)	6189 (61%)	459 (52%)	
Diabetes, *n* (%)				< 0.001
No	11,602 (92%)	10,748 (92%)	854 (86%)	
Yes	1466 (8%)	1296 (7%)	170 (14%)	
Cardiovascular disease, *n* (%)				< 0.001
No	11,688 (92%)	10,891 (93%)	797 (79%)	
Yes	1380 (8%)	1153 (7%)	227 (21%)	
Smoking status, *n* (%)				0.546
No	6768 (52%)	6236 (52%)	532 (51%)	
Yes	6300 (48%)	5808 (48%)	492 (49%)	
Alcohol consumption, *n* (%)				< 0.001
No	3517 (22%)	3065 (21%)	452 (38%)	
Yes	9551 (78%)	8979 (79%)	572 (62%)	
PIR	3.29 (1.68, 5.00)	3.37 (1.71, 5.00)	2.52 (1.46, 4.55)	< 0.001
PA, *n* (%)				< 0.001
No	7601 (54%)	6887 (53%)	714 (67%)	
Yes	5467 (46%)	5157 (47%)	310 (33%)	
Creatinine, mg/dL	0.89 (0.75, 1.00)	0.90 (0.75, 1.00)	0.82 (0.72, 1.00)	< 0.001
Blood urea nitrogen, mg/dL	13.00 (10.00, 16.00)	12.00 (10.00, 15.00)	14.00 (11.00, 18.00)	< 0.001
GGT, U/L	20.00 (14.00, 30.00)	20.00 (14.00, 30.00)	18.00 (13.00, 28.00)	< 0.001
25‐Hydroxyvitamin D, nmol/L	67.00 (51.00, 82.00)	66.00 (51.00, 82.00)	73.00 (54.00, 90.00)	< 0.001
Serum calcium, mg/dL	9.40 (9.20, 9.70)	9.40 (9.20, 9.70)	9.50 (9.20, 9.70)	0.374
Femoral neck BMD, g/cm^2^	0.82 (0.73, 0.92)	0.83 (0.74, 0.93)	0.62 (0.54, 0.71)	< 0.001
ePWV, m/s	7.67 (6.55, 9.36)	7.53 (6.50, 9.11)	10.12 (8.48, 12.02)	< 0.001

Abbreviations: BMD, bone mineral density; BMI, body mass index; ePWV, estimated pulse wave velocity; GGT, gamma‐glutamyl transferase; PA, physical activity.

Compared with participants without osteoporosis, those with osteoporosis were more likely to be female and older, with a higher proportion of non‐Hispanic White individuals. They were more frequently widowed, divorced, or separated and exhibited lower socioeconomic status and lower participation in PA. From a metabolic perspective, participants with osteoporosis had a higher prevalence of cardiovascular disease and diabetes and higher levels of blood urea nitrogen and serum 25‐hydroxyvitamin D, whereas BMI, serum creatinine, and GGT levels were relatively lower. No significant difference in total serum calcium levels was observed between the two groups.

Regarding bone health indicators, femoral neck BMD was significantly lower in the osteoporosis group, and ePWV levels were markedly higher compared with those in the nonosteoporosis group (all *p* < 0.05).

### 3.2. Association Between ePWV and Bone Health Outcomes

Table 2 presents the results of multivariable analyses examining the associations of ePWV with the prevalence of osteoporosis and femoral neck BMD. In the fully adjusted model (Model 3), ePWV was positively associated with prevalent osteoporosis (OR = 1.223, 95% CI: 1.136–1.318, *p* < 0.001) and inversely associated with femoral neck BMD (*β* = −0.030, 95% CI: −0.035, −0.024, *p* < 0.001).

**TABLE 2:** Associations of ePWV with prevalent osteoporosis and femoral neck BMD.

Category	Model 1 (effect, 95% CI)	*p*	Model 2 (effect, 95% CI)	*p*	Model 3 (effect, 95% CI)	*p*
Osteoporosis						
ePWV	1.302 (1.223, 1.386)	< 0.001	1.244 (1.163, 1.331)	< 0.001	1.223 (1.136, 1.318)	< 0.001
T1 (< 7.036)	Ref.		Ref.		Ref.	
T2 (7.036–9.344)	1.742 (1.160, 2.618)	0.010	2.000 (1.342, 2.979)	0.001	1.980 (1.325, 2.960)	0.002
T3 (> 9.344)	3.297 (2.123, 5.119)	< 0.001	3.346 (2.115, 5.292)	< 0.001	3.235 (2.015, 5.194)	< 0.001
*p* for trend	< 0.001		< 0.001		< 0.001	
Femoral neck BMD						
ePWV	−0.030 (−0.035, −0.024)	< 0.001	−0.031 (−0.036, −0.026)	< 0.001	−0.030 (−0.035, −0.024)	< 0.001
T1 (< 7.036)	Ref.		Ref.		Ref.	
T2 (7.036–9.344)	−0.016 (−0.026, −0.005)	0.005	−0.034 (−0.044, −0.025)	< 0.001	−0.034 (−0.043, −0.025)	< 0.001
T3 (> 9.344)	−0.049 (−0.064, −0.034)	< 0.001	−0.062 (−0.076, −0.048)	< 0.001	−0.059 (−0.074, −0.045)	< 0.001
*p* for trend	< 0.001		< 0.001		< 0.001	

*Note:* Model 1 was adjusted for age, sex, and race. Model 2 was further adjusted for PIR, marital status, educational status, BMI, and PA. Model 3 was fully adjusted for age, sex, race, PIR, marital status, educational status, BMI, PA, smoking status, alcohol consumption, diabetes, cardiovascular disease, serum creatinine, GGT, blood urea nitrogen, 25‐hydroxyvitamin D, and serum calcium.

Abbreviations: BMD, bone mineral density; BMI, body mass index; CI, confidence interval; ePWV, estimated pulse wave velocity; GGT, gamma‐glutamyl transferase; PA, physical activity; PIR, poverty–income ratio.

After excluding participants with ePWV values below the 1st percentile or above the 99th percentile, the results remained largely unchanged (Supporting Table S1). In Model 3, higher ePWV remained significantly associated with higher odds of prevalent osteoporosis (OR = 1.260, 95% CI: 1.161, 1.366, *p* < 0.001) and lower femoral neck BMD (*β* = −0.030, 95% CI: −0.036, −0.025, *p* < 0.001).

### 3.3. Nonlinear Associations Between ePWV and Bone Health Outcomes

**FIGURE 2:**
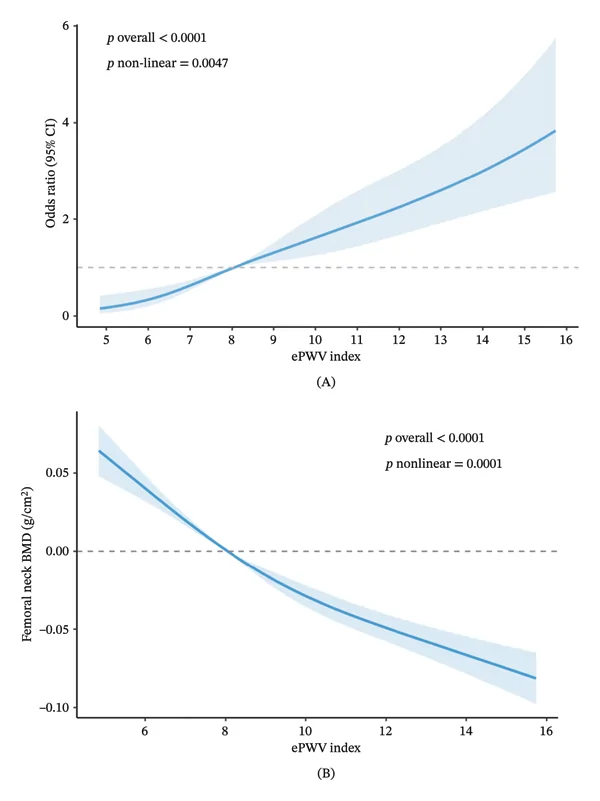
RCS analyses of the associations between ePWV and bone health outcomes. (A) Nonlinear association between ePWV and the odds of prevalent osteoporosis. (B) Nonlinear association between ePWV and femoral neck bone mineral density. Abbreviations: ePWV, estimated pulse wave velocity; RCS, restricted cubic spline; BMD, bone mineral density; OR, odds ratio; CI, confidence interval.

RCS analyses revealed a significant nonlinear association between ePWV and the odds of prevalent osteoporosis (Figure 2A). Threshold effect analysis further identified an inflection point at an ePWV value of 8.39 (Table 3). When ePWV was ≤ 8.39, the odds of prevalent osteoporosis increased markedly with rising ePWV (OR = 1.777, 95% CI: 1.467, 2.153, *p* < 0.001). When ePWV exceeded 8.39, the odds continued to increase, although the magnitude of the association was substantially attenuated (OR = 1.168, 95% CI: 1.106, 1.234, *p* < 0.001). Likelihood ratio tests indicated that the segmented regression model provided a significantly better fit than the single linear model (*p* < 0.001).

**TABLE 3:** Threshold effect analyses of the associations between ePWV and bone health outcomes.

Outcome	Inflection point (*K*)	ePWV < *K*	ePWV > *K*	*p* for likelihood ratio test
Osteoporosis (OR, 95% CI)	8.393	1.777 (1.467, 2.153)	1.168 (1.106, 1.234)	< 0.001
Femoral neck BMD *β* (95% CI)	8.591	−0.021 (−0.025, −0.017)	−0.010 (−0.016, −0.004)	< 0.001

Abbreviations: BMD, bone mineral density; CI, confidence interval; ePWV, estimated pulse wave velocity; OR, odds ratio.

Similarly, a significant nonlinear relationship was observed between ePWV and femoral neck BMD (Figure 2B). Threshold analysis identified an inflection point at an ePWV value of 8.591 (Table 3). When ePWV was ≤ 8.591, femoral neck BMD decreased significantly with increasing ePWV (*β* = −0.021, 95% CI: −0.025, −0.017, *p* < 0.001). Beyond this threshold (ePWV > 8.591), the decline in femoral neck BMD persisted but at a slower rate (*β* = −0.010, 95% CI: −0.016, −0.004, *p* < 0.001).

### 3.4. Subgroup Analyses

**FIGURE 3:**
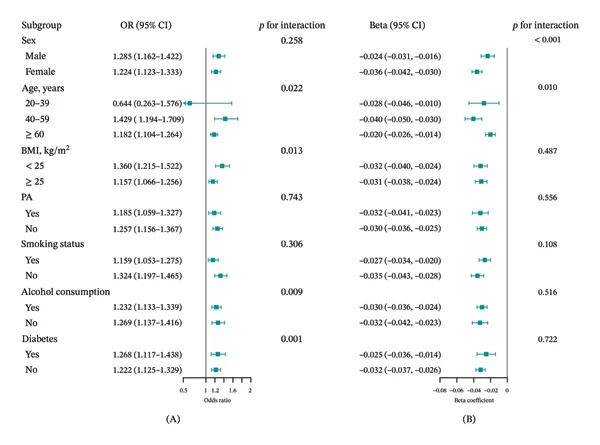
Subgroup analyses illustrating the associations between ePWV and bone health outcomes across different population characteristics. (A) Association between ePWV and osteoporosis. (B) Association between ePWV and femoral neck bone mineral density. Abbreviations: BMI, body mass index; PA, physical activity; ePWV, estimated pulse wave velocity.

Subgroup analyses demonstrated that the associations between ePWV and bone health outcomes were generally consistent across most subgroups (Figure 3). For osteoporosis (Figure 3A), the positive association between higher ePWV and higher odds of prevalent osteoporosis was largely consistent across strata defined by sex, PA, and smoking status. However, significant interactions were observed across subgroups stratified by age, BMI, alcohol consumption, and diabetes status (*p* for interaction < 0.05).

Regarding femoral neck BMD (Figure 3B), ePWV was inversely associated with BMD across all examined subgroups. Significant effect modification was identified in analyses stratified by sex and age (*p* for interaction < 0.05), whereas no statistically significant interactions were observed for the remaining subgroup variables.

### 3.5. The ROC Analysis of ePWV and Osteoporosis

**FIGURE 4:**
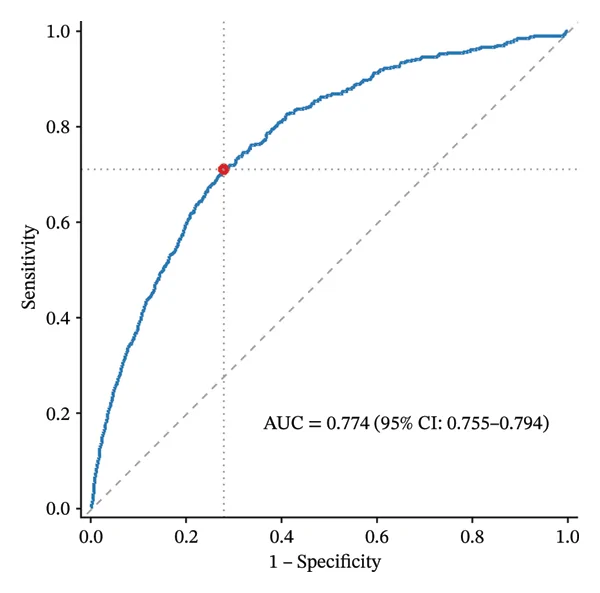
Weighted receiver operating characteristic curve of ePWV for osteoporosis. Abbreviations: ePWV, estimated pulse wave velocity; ROC, receiver operating characteristic; AUC, area under the ROC curve.

Weighted ROC analysis showed that ePWV had moderate discriminative ability in identifying prevalent osteoporosis (Table 4 and Figure 4), with an AUC of 0.774 (95% CI: 0.755, 0.794, *p* < 0.001). The optimal cutoff value for ePWV was 8.865 m/s. This cutoff yielded a sensitivity of 0.711, specificity of 0.721, positive predictive value of 0.159, and negative predictive value of 0.971.

**TABLE 4:** Weighted receiver operating characteristic analysis of ePWV for osteoporosis.

AUC (95% CI)	*p*‐value	Cutoff value	Youden index	Sensitivity	Specificity	PPV	NPV
0.774 (0.755–0.794)	< 0.001	8.865	0.432	0.711	0.721	0.159	0.971

*Note:* AUC, area under the ROC curve.

Abbreviations: ePWV, estimated pulse wave velocity; NPV, negative predictive value; PPV, positive predictive value; ROC, receiver operating characteristic.

## 4. Discussion

Using nationally representative data from NHANES, this study systematically evaluated the associations between the vascular aging index ePWV and two key bone health outcomes, osteoporosis, and bone mineral density. Multivariable analyses demonstrated that higher ePWV levels were consistently associated with higher odds of prevalent osteoporosis and lower femoral neck BMD after stepwise adjustment for potential confounders. Furthermore, RCS analyses revealed significant nonlinear relationships between ePWV and bone health outcomes, characterized by identifiable inflection points. The overall pattern suggested a steeper association at lower ePWV levels, followed by a more gradual slope at higher levels. Subgroup analyses indicated that these associations were directionally consistent across most population subgroups, with effect heterogeneity observed only in selected characteristics, supporting the overall robustness of the findings.

From a biological perspective, vascular aging may be associated with impaired bone health through multiple interrelated pathways. Increased arterial stiffness is characterized by reduced vascular compliance and altered hemodynamic properties, which may be related to impaired microvascular perfusion within bone tissue [26, 27]. Bone is a highly metabolically active tissue that depends on adequate blood supply to maintain the dynamic balance between bone formation and resorption [28]. When microcirculatory perfusion is compromised, insufficient delivery of oxygen and nutrients to osteoblasts may inhibit their differentiation and function, while osteoclast activity may become relatively enhanced, thereby accelerating bone loss [29, 30]. In addition, vascular aging is commonly accompanied by chronic low‐grade inflammation and oxidative stress [31]. Persistently elevated inflammatory mediators and reactive oxygen species can disrupt signaling pathways involved in bone remodeling, impair bone matrix turnover, and accelerate deterioration of bone microarchitecture.

The threshold effect observed in this study should be interpreted cautiously. Below the identified inflection points, namely, 8.393 m/s for osteoporosis and 8.591 m/s for femoral neck BMD, increases in ePWV were associated with a steeper rise in osteoporosis risk and a more pronounced decline in BMD. This pattern may suggest that early arterial stiffening is particularly relevant to bone microcirculatory perfusion, endothelial function, and oxygen and nutrient delivery, thereby disturbing the balance between bone formation and resorption. Above these thresholds, the associations remained statistically significant but became less steep. This attenuation may reflect a saturation effect, a lower residual range of femoral neck BMD among high‐risk individuals, or the accumulation of age‐related and metabolic comorbidities that independently affect bone metabolism. Similar threshold or nonlinear patterns have been reported for arterial stiffness in relation to cardiovascular target‐organ damage and chronic kidney disease, supporting the plausibility of a threshold‐based interpretation [32, 33]. It should be noted that these thresholds should be interpreted as statistical inflection points reflecting changes in the strength of association, rather than as diagnostic cutoffs for osteoporosis. Further prospective studies are needed to determine whether these threshold values can be used for bone health risk stratification in clinical practice.

Beyond impaired perfusion, endothelial dysfunction, chronic inflammation, and oxidative stress may jointly disturb bone homeostasis and contribute to the observed associations between ePWV and bone health. Vascular and skeletal tissues are closely interconnected in the regulation of calcium–phosphorus homeostasis. During vascular calcification, vascular smooth muscle cells undergo osteogenic transdifferentiation, and the molecular regulators involved in calcium–phosphate deposition substantially overlap with those governing bone remodeling, jointly modulating the balance between bone resorption and formation [34, 35]. Vitamin D–related pathways represent another critical link. The active form of vitamin D regulates intestinal calcium absorption and osteocyte differentiation, while vitamin D deficiency has been associated with endothelial dysfunction and arterial stiffness. Through reduced nitric oxide production and enhanced inflammatory responses, vitamin D insufficiency may further disrupt the bone microenvironment [36, 37]. Moreover, paracrine factors secreted by endothelial cells, such as vascular endothelial growth factor (VEGF) and endothelial‐derived growth factors, not only regulate local blood flow and bone perfusion but also directly influence osteoblast and osteoclast activity. Impaired endothelial function may disrupt these signaling pathways, ultimately weakening bone formation capacity [38]. These mechanisms do not operate in isolation but rather interact synergistically, underscoring the multifaceted role of vascular aging in bone health deterioration.

The effect heterogeneity observed across specific subgroups suggests that the association between vascular aging and bone health may be modified by population characteristics. Age is closely related to both vascular and skeletal health. With advancing age, arterial elasticity tends to decline, and vascular aging becomes more evident, while bone mineral loss also becomes more pronounced. This shared aging trajectory may strengthen the link between vascular dysfunction and bone loss, particularly among older adults [39, 40]. Sex‐related differences may be partly explained by hormonal factors, especially in women [41]. Declining estrogen levels are associated with both bone loss and arterial stiffening, which may partly account for the closer coupling between vascular dysfunction and skeletal deterioration in female populations [42]. Body weight may modulate bone health through mechanical loading. Individuals with higher BMI may partially preserve bone mass due to increased skeletal loading, whereas those with lower BMI often have reduced baseline bone reserves and greater dependence on adequate blood perfusion and nutrient supply. Consequently, vascular dysfunction may be more closely associated with impaired bone health in individuals with lower BMI [43]. In individuals with diabetes, chronic metabolic abnormalities and endothelial dysfunction may represent key mechanisms linking vascular aging to impaired bone health [44]. Hyperglycemia not only accelerates arterial stiffening but also disrupts bone remodeling and repair, suggesting a more complex vascular–skeletal relationship in individuals with diabetes [45]. Differences observed across alcohol consumption strata suggest that alcohol may influence the vascular–bone axis through multiple pathways. Chronic or excessive alcohol intake can impair endothelial function and reduce arterial elasticity, which may be related to vascular aging and impaired bone perfusion and nutrient delivery [46]. Concurrently, alcohol may suppress sex hormone levels and increase inflammatory activity, disrupting bone metabolic balance by reducing osteogenesis and enhancing bone resorption [47]. Together, these mechanisms may amplify or modify the effects of vascular aging on bone health.

### 4.1. Clinical Implications

From a clinical perspective, ePWV may serve as a convenient auxiliary marker for preliminary bone health risk stratification. Since ePWV is calculated using routinely available age and blood pressure data, it is noninvasive, inexpensive, and does not require specialized equipment, which supports its potential use in population‐based health examinations and primary care settings. The threshold range identified in this study, approximately 8.393–8.591 m/s, may help identify individuals who require closer attention to skeletal health, particularly older adults, women, individuals with low BMI, and those with metabolic risk factors such as diabetes. For individuals above this range, further evaluation of osteoporosis‐related risk factors and DXA measurement may be considered when clinically indicated. However, ePWV should be regarded only as an auxiliary risk‐stratification marker rather than a diagnostic tool. Because it is derived from age and blood pressure, ePWV reflects vascular aging and hemodynamic burden, but it cannot directly assess bone mass or replace DXA‐based BMD measurement. Its main clinical value may lie in helping identify individuals who could benefit from earlier or more frequent bone health assessment, rather than independently diagnosing osteoporosis.

### 4.2. Strengths and Limitations

This study has several notable strengths. First, it was based on a large, nationally representative sample, enhancing the generalizability of the findings. Second, both osteoporosis and BMD were evaluated, allowing for a comprehensive assessment of bone health from both categorical and continuous perspectives. Third, a stepwise multivariable adjustment strategy was applied to control for a wide range of potential confounders. Importantly, nonlinear analytical approaches were used to identify dose–response patterns that may be overlooked by conventional linear models. In addition, sensitivity analyses excluding participants with extreme ePWV values further confirmed the robustness of the primary findings. Finally, the simplicity and accessibility of ePWV support its potential value in large‐scale screening and preliminary risk stratification for impaired bone health.

Several limitations should also be acknowledged. First, the cross‐sectional design precludes determination of causal relationships or temporal sequencing between vascular aging and bone health outcomes. Prospective studies are, therefore, needed to confirm these associations. Second, part of the osteoporosis information was based on self‐reported physician diagnoses, which may introduce recall bias or misclassification. Third, ePWV is an estimated measure derived from age and blood pressure and, although suitable for population‐based research, cannot fully replace directly measured vascular function indices. Finally, despite extensive covariate adjustment, residual confounding from unmeasured factors cannot be entirely excluded. Future studies integrating longitudinal designs with direct vascular assessments and more detailed bone phenotyping are warranted to further clarify the relationship between vascular aging and bone health.

## 5. Conclusion

In conclusion, this NHANES‐based study suggests that the vascular aging index ePWV is nonlinearly associated with osteoporosis and BMD, with generally robust associations across most population subgroups. Prospective cohort studies incorporating direct measures of vascular function and more objective assessments of bone health are needed to validate these findings and elucidate the underlying mechanisms.

## Funding

The authors have nothing to report.

## Conflicts of Interest

The authors declare no conflicts of interest.

## Supporting Information

Additional supporting information can be found online in the Supporting Information section.

## Supporting information


**Supporting Information** Table S1 presents the associations of estimated pulse wave velocity (ePWV) with osteoporosis and femoral neck bone mineral density (BMD). Multivariate logistic and linear regression analyses were performed across three gradually adjusted models. Model 1 adjusted for age, sex, and race; Model 2 additionally controlled for socioeconomic and lifestyle factors; Model 3 was the fully adjusted model incorporating multiple biochemical indicators and chronic diseases. The results demonstrated that elevated ePWV was significantly associated with higher odds of osteoporosis and lower femoral neck BMD.

## Data Availability

The data and materials in the current study are available from the corresponding author on reasonable request.
