# Supplementary material for: Nonlinear Association of Estimated Pulse Wave Velocity With Bone Health Outcomes: Evidence From the NHANES Database
Source: Int J Endocrinol. 2026 Aug 28;2026:4794146. doi: 10.1155/ije/4794146 (PMC13523602; doi:10.1155/ije/4794146)
Supplement: Supplementary file 1 [file IJE-2026-4794146-s001.docx]

**Table S1. Associations of ePWV with osteoporosis and femoral neck BMD**

| **Category** | **Model 1** | | **Model 2** | | **Model 3** | |
| --- | --- | --- | --- | --- | --- | --- |
|  | **Effect (95% CI)** | **P** | **Effect (95% CI)** | **P** | **Effect (95% CI)** | **P** |
| **Osteoporosis** | | | | | | |
| ePWV | 1.341 (1.249, 1.440) | <0.001 | 1.280 (1.187, 1.380) | <0.001 | 1.260 (1.161, 1.366) | <0.001 |
| T1 (<7.051) | Ref. |  | Ref. |  | Ref. |  |
| T2 (7.051-9.316) | 1.712 (1.107, 2.648) | 0.019 | 1.963 (1.278, 3.015) | 0.003 | 1.940 (1.259, 2.991) | 0.005 |
| T3 (>9.316) | 3.307 (2.064, 5.297) | <0.001 | 3.382 (2.058, 5.560) | <0.001 | 3.286 (1.960, 5.509) | <0.001 |
| P for trend |  | <0.001 |  | <0.001 |  | <0.001 |
| **Femoral neck BMD** | | | | | | |
| ePWV | -0.030 (-0.035, -0.024) | <0.001 | -0.032 (-0.037, -0.026) | <0.001 | -0.030 (-0.036, -0.025) | <0.001 |
| T1 (<7.051) | Ref. |  | Ref. |  | Ref. |  |
| T2 (7.051-9.316) | -0.016 (-0.026, -0.005) | 0.004 | -0.033 (-0.043, -0.024) | <0.001 | -0.033 (-0.042, -0.024) | <0.001 |
| T3 (>9.316) | -0.047 (-0.062, -0.031) | <0.001 | -0.061 (-0.075, -0.046) | <0.001 | -0.058 (-0.073, -0.044) | <0.001 |
| P for trend |  | <0.001 |  | <0.001 |  | <0.001 |

Model 1 was adjusted for age, sex, and race.

Model 2 was further adjusted for PIR, marital status, educational status, BMI, and PA.

Model 3 was fully adjusted for age, sex, race, PIR, marital status, educational status, BMI, PA, smoking status, alcohol consumption, diabetes, cardiovascular disease, serum creatinine, GGT, blood urea nitrogen, 25-hydroxyvitamin D, and serum calcium.

Abbreviations: ePWV, estimated pulse wave velocity; PIR, poverty-income ratio; BMI, body mass index; PA, physical activity; BMD, bone mineral density; GGT, gamma-glutamyl transferase; CI, confidence interval.

.
